# FedMIR: Multimodal Federated Learning with Missing Modality Imputation and Distribution-Aware Routing

**DOI:** 10.3390/s26102954

**Published:** 2026-05-08

**Authors:** Hongyu Xiong, Ming Dai

**Affiliations:** 1 Haide College, Ocean University of China, Qingdao 266100, China; xionghongyu@stu.ouc.edu.cn; 2 School of Mathematics and Computer, Guangdong Ocean University, Zhanjiang 524008, China

**Keywords:** multimodal federated learning, internet of things, mixture of experts

## Abstract

Existing multimodal federated learning methods typically assume complete modality availability and struggle with heterogeneity between training and testing data distributions, making them unsuitable for handling missing modalities and distribution drift in distributed learning scenarios such as the Internet of Things (IoT). To address these challenges, we present FedMIR, a novel framework for multimodal federated learning. Our key observation is that heterogeneous modalities can be mapped into a shared semantic space, where cross-modal dependencies can be effectively modeled. Based on this insight, FedMIR leverages contrastive learning to align image–text modalities in a shared latent space and employs conditional generation to reconstruct missing modality representations. The completed representations are then routed through a mixture-of-experts backbone conditioned on the estimated distribution state. FedMIR shares only model parameters and distribution statistics with the server. This design enables the model to operate under missing modality settings while adaptively allocating expert knowledge to cope with distribution drift. We validate FedMIR on federated image–text retrieval benchmarks under heterogeneity and missing data conditions, demonstrating its effectiveness compared to representative federated learning baselines.

## 1. Introduction

In recent years, the explosive growth of Internet of Things (IoT) deployments has promoted the application of multimodal deep learning in various fields, ranging from smart homes and healthcare to transportation and industrial monitoring [1,2,3,4,5,6,7,8]. These IoT data are generated and dispersed across mobile devices and edge gateways. Due to privacy regulations and network constraints, such data cannot usually be shared directly, resulting in isolated data silos [9]. Furthermore, IoT devices are often produced by different manufacturers, deployed in diverse environments, and equipped with heterogeneous hardware, leading to significant data heterogeneity. Environmental unpredictability, including occlusion, low illumination, and network instability, further introduces noise and leads to missing data [10,11]. Multimodal models typically rely on large-scale, fully available data to learn cross-modal distribution patterns and adapt to downstream tasks. As a result, training effective multimodal models becomes challenging when data are isolated and distributed at the edge [12].

Multimodal federated learning (MFL) provides a distributed learning framework that enables clients to train local models on edge devices while collaboratively learning a global multimodal model by sharing only model parameters instead of raw data [12,13]. This paradigm effectively protects user privacy while facilitating knowledge sharing. Since data distributions vary across modalities, existing MFL methods primarily focus on aligning features from different modalities for effective fusion. Such alignment establishes a mapping between multimodal data representations and task objectives [14].

Existing MFL approaches are commonly categorized according to the fusion stage into late fusion, intermediate fusion, and hierarchical fusion [13,14]. In late fusion, the central server directly aggregates the predicted probabilities output by clients at the final model layer to combine knowledge from multiple sources [15,16,17,18]. However, in federated learning scenarios, client data are highly heterogeneous. Consequently, the predicted probabilities of a single client model only reflect its adaptation to a specific data distribution. When confronted with unseen distributions, such models may produce incorrect predictions with high confidence, leading to erroneous information being incorporated during aggregation and ultimately degrading global model performance. Intermediate fusion methods train modality-specific encoders on clients and project their representations into a shared latent space, where aligned features are fused for downstream tasks [19,20,21,22,23,24]. Nevertheless, due to distributional discrepancies among modalities, directly fusing heterogeneous representations may introduce knowledge conflicts and negatively impact model performance. Hierarchical fusion methods address this issue by adaptively weighting features from different modalities to mitigate conflicts caused by distributional imbalance [25,26,27,28,29]. However, in IoT environments, clients often suffer from missing modalities, resulting in incomplete modal features. This limitation prevents certain clients from performing multimodal fusion, thereby restricting the applicability of hierarchical fusion methods.

**Figure 1:**
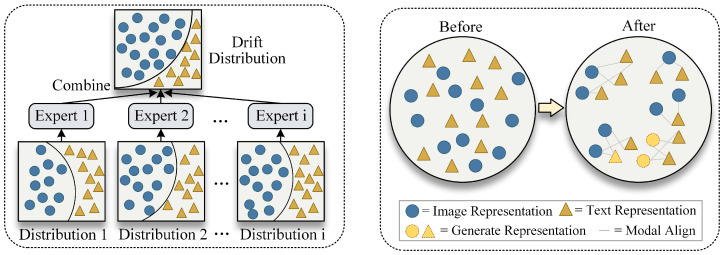
Illustration of challenges in IoT multimodal federated learning. (**a**) Distribution-aware dynamic routing: heterogeneous clients have different local data distributions, and a mixture-of-experts architecture combines specialized experts to handle distribution drift across clients. (**b**) Contrastive inverse generation: before alignment, image representations (blue circles) and text representations (gold triangles) are scattered without cross-modal correspondence; after contrastive alignment, paired representations are pulled together in a shared semantic space, and missing modality representations (dashed gold triangles) are conditionally generated from observed ones.

As illustrated in Figure 1, we consider a real-world IoT scenario where clients are equipped with heterogeneous sensors and experience varying environmental conditions. Therefore, we cannot assume complete modality availability on all clients, nor can we assume stationary data distributions throughout the deployment lifecycle. Existing MFL methods rely on modality integrity and client distribution consistency, meaning that they are no longer applicable in this context.

Our key observation is that, during the representation learning process, different modalities describing the same semantic entity should produce similar representations in a shared latent space. As a result, we can leverage cross-modal semantic consistency to infer missing modality representations from the observed ones. Although the basic idea is intuitive, FedMIR faces two significant challenges: First, the statistical properties of different modalities vary considerably, making it difficult to infer the characteristics of unobserved modalities from heterogeneous observed signals. Second, in practice, environmental conditions, user behaviors, and device characteristics can change over time in ways that cannot be fully anticipated during training. This leads to a decrease in model performance.

To address these challenges, we propose **FedMIR** for multimodal federated learning in IoT settings with missing modalities and distribution shifts. Specifically, we introduce contrastive inverse generation (CIG), which first aligns modality-specific encoders via a contrastive objective. Then, a conditional generator learns to infer the representation of missing modalities from the observed ones, allowing downstream models to work without requiring complete data transmission. Additionally, we propose distribution-aware dynamic routing (DADR), which augments a mixture-of-experts (MoE) backbone with lightweight distribution state estimation. DADR dynamically adjusts the number of activated experts based on the detected drift level, without requiring prior knowledge of specific shift types or magnitudes. Throughout this paper, all inter-client communication is restricted to model parameter updates; neither raw sensor observations nor intermediate feature embeddings are ever transmitted across clients or exposed to the server. Our major contributions are as follows:

- We present a novel framework for MFL under missing modalities and distribution shifts. We demonstrate for the first time how cross-modal semantic consistency and distribution state estimation can be jointly leveraged to address these challenges in IoT-oriented MFL.
- We design CIG, which aligns heterogeneous modalities via a contrastive objective in a shared latent space and performs conditional semantic generation to recover missing modality representations on clients. This enables downstream models to consume unified multimodal inputs.
- We introduce DADR, which combines distribution state monitoring with distribution-aware gating and dynamic expert activation, improving the robustness of MoE backbones under dynamic IoT conditions.
- We extensively evaluate FedMIR on two widely used multimodal retrieval benchmarks. The results validate the effectiveness of FedMIR, showing that it outperforms existing MFL methods under various missing ratios and maintains robust performance across different client configurations.

The remainder of this paper is organized as follows: Section 2 gives an overview of related works. Section 3 formulates the problem. Section 4 details the design of FedMIR and is followed by extensive performance evaluation in Section 5. Finally, Section 6 concludes the paper.

## 2. Related Work

We begin by reviewing MFL methods through the lens of fusion stages, i.e., late fusion, intermediate fusion, and hierarchical fusion [13,14], then discuss MoE architectures and their generalization behavior under distribution shifts. Table 1 summarizes the key characteristics of different method categories.

### 2.1. Late Fusion MFL

Late fusion methods keep training entirely on clients and aggregate only the prediction outputs or soft labels on the server. FedMD [15] distills client logits on a public proxy dataset so that heterogeneous models can collaborate without sharing weights. FedDF [16] further removes the requirement for public data by using a generator to provide synthetic samples for distillation. FedGEMS [17] builds a larger server model through selective knowledge fusion from multiple clients and uses this enhanced model to guide client training in subsequent rounds. FedMultiEmo [18] aggregates emotion predictions from multiple modalities, including audio, visual, and physiological signals. However, the performance of late fusion methods is constrained by the decision quality of each local model. Under missing modalities and data heterogeneity, local models may produce highly divergent predictions, making it difficult for the aggregated global model to approach an optimal solution.

### 2.2. Intermediate Fusion MFL

Intermediate fusion methods learn modality-specific encoders on clients using local multimodal data, then fuse the resulting representations on the server or through coordinated learning. For cross-modal retrieval tasks, FedVMR [19] operates under decentralized data by aligning video and text representations across clients. FedCola [20] adapts vision and language transformers to MFL through parameter sharing and knowledge exchange mechanisms, which narrows both intra-modality and cross-modality representation gaps. For medical applications, Incongruent MFL [21] addresses the challenge of heterogeneous medical imaging and clinical text data across healthcare institutions. FedAF [22] includes alignment-augmented fusion for federated multimodal learning scenarios with limited labeled data. Yin et al. [23] leveraged knowledge distillation to avoid task conflicts in cross-modal federated learning. Ma et al. [24] incorporated differential privacy mechanisms with modality selection to protect sensitive information while maintaining multimodal learning performance. However, missing modalities remain common in IoT deployments and can introduce large distribution gaps among embeddings produced by different clients. Directly fusing these heterogeneous representations without proper alignment may lead to knowledge conflicts and degrade global model performance.

### 2.3. Hierarchical Fusion MFL

Hierarchical fusion methods assign dynamic weights to features from different modalities and clients so that higher-quality features contribute more to the final prediction while low-quality or missing modality inputs are down-weighted. MMFed [25] is a multimodal federated learning framework which adaptively balances the contributions from different modalities according to their quality and availability. Zhang et al. [26] introduced a cross-modal federated learning approach with hierarchical aggregation that enables unimodal training on clients while supporting multimodal prediction at the server. Yin et al. [27] combined self-attention fusion with adaptive continual updating to handle heterogeneous multimodal data streams in federated settings. ModiFedCat [28] comprises a multimodal distillation-based federated catalytic framework. Tun et al. [29] proposed resource-efficient federated multimodal learning through layer-wise and progressive training strategies. However, learning and applying these adaptive weights incurs additional computational overhead, which may be prohibitive for resource-constrained IoT devices. Since client data are highly heterogeneous, the learned weights can differ across clients, causing large fluctuations in the aggregated information and making it harder for the global model to converge.

### 2.4. Mixture of Experts and Distribution Generalization

MoE architectures improve model capacity and robustness under distribution shifts by decomposing the model into multiple specialized experts and learning a router that selects experts according to input characteristics. Uni-MoE [30] scales unified multimodal large language models with mixture of experts. Zhuang et al. [31] applied MoE to personalized federated learning for fault diagnosis, where different experts capture client-specific patterns while sharing common knowledge. For vision–language tasks, FedVLA [32] combines vision–language alignment with expert routing in federated settings. DFLMoE [33] and FedDG [34] further explore MoE architectures for domain generalization in decentralized learning scenarios. Despite these advances, most MoE-based generalization methods are developed in centralized settings with stability assumptions. They do not directly address the unique challenges of multimodal federated IoT deployments, including bimodal missing modality patterns across clients and continuous distribution drift driven by dynamic environmental conditions.

By contrast, FedMIR is a new framework that integrates cross-modal semantic alignment, missing modality completion, and distribution-aware adaptive routing for multimodal federated learning under practical heterogeneity conditions. Its CIG module is inspired by intermediate fusion approaches that align modality-specific representations in a shared semantic space; however, it explicitly recovers missing modality representations through conditional generation rather than merely fusing available ones. Furthermore, the routing decision is made dependent on a continuously estimated distribution state rather than static labels.

## 3. Preliminaries

Multimodal federated learning provides a distributed training paradigm for IoT systems, where distributed clients [12] collaborate to learn a shared model. In this paper, we focus on federated image–text retrieval, where each sample is an image–text pair rather than a class-labeled example. We consider a central server and a set of clients C={1,…,C}. Each client c∈C corresponds to an IoT device that continuously collects local multimodal data in its deployment environment $e_{c}\in E$. Let M={I,T} denote the image and text modalities. Client *c* may observe one or both modalities depending on its hardware configuration and operational status. The local dataset on client *c* is defined as

(1) $$D_{c}={\left\{\left(x_{i}^{I},x_{i}^{T},r_{i}^{I},r_{i}^{T}\right)\right\}}_{i=1}^{n_{c}}$$

where $x_{i}^{I}$ and $x_{i}^{T}$ denote the image and text paired with the same semantic entity, and $r_{i}^{I},r_{i}^{T}\in \left\{0,1\right\}$ are availability indicators for the two modalities of sample *i*. The availability indicators model missing modalities caused by device heterogeneity, sensor failures, and communication constraints. Our goal is to learn a mapping f(·;θ) that transforms the available image–text observations on each client into aligned multimodal representations for bidirectional retrieval.

For a mini batch $B_{c}={\left\{\left(x_{p}^{I},x_{p}^{T}\right)\right\}}_{p=1}^{B}$ sampled from client *c*, let $z_{p}^{I}$ and $z_{p}^{T}$ denote the image and text embeddings produced by f(·;θ) in the shared semantic space after processing the available modalities. We define the scaled cosine similarity between image *p* and text *q* as

(2) $$s_{pq}=\frac{\mathrm{sim}(z_{p}^{I},z_{q}^{T})}{\tau },$$

where sim(·,·) is cosine similarity and τ>0 is a temperature parameter. The image-to-text retrieval loss and the symmetric text-to-image retrieval loss are given by

(3) $$L_{i2t}=-\frac{1}{B}\sum_{p=1}^{B}\log\frac{\exp(s_{pp})}{\sum_{q=1}^{B}\exp\left(s_{pq}\right)},$$

(4) $$L_{t2i}=-\frac{1}{B}\sum_{p=1}^{B}\log\frac{\exp(s_{pp})}{\sum_{q=1}^{B}\exp\left(s_{qp}\right)},$$

and the batch retrieval loss is

(5) $$\ell_{\mathrm{ret}}\left(B_{c};\theta \right)=\frac{1}{2}\left(L_{i2t}+L_{t2i}\right).$$

Accordingly, the task-dependent loss *ℓ* in our problem setting is instantiated as $\ell_{\mathrm{ret}}$, and the local empirical risk on client *c* is

(6) $$F_{c}\left(\theta \right)=E_{B_{c}∼D_{c}}\left[\ell_{\mathrm{ret}}\left(B_{c};\theta \right)\right].$$

Federated training aims to find a shared parameter θ that minimizes the weighted sum of local objectives

(7) $$F\left(\theta \right)=\sum_{c\in C}\frac{n_{c}}{N}F_{c}\left(\theta \right),\;N=\sum_{c\in C}n_{c},$$

so that the global model learns cross-modal retrieval knowledge from heterogeneous client datasets while keeping all raw image–text pairs on local devices.

## 4. Design of FedMIR

**Figure 2:**
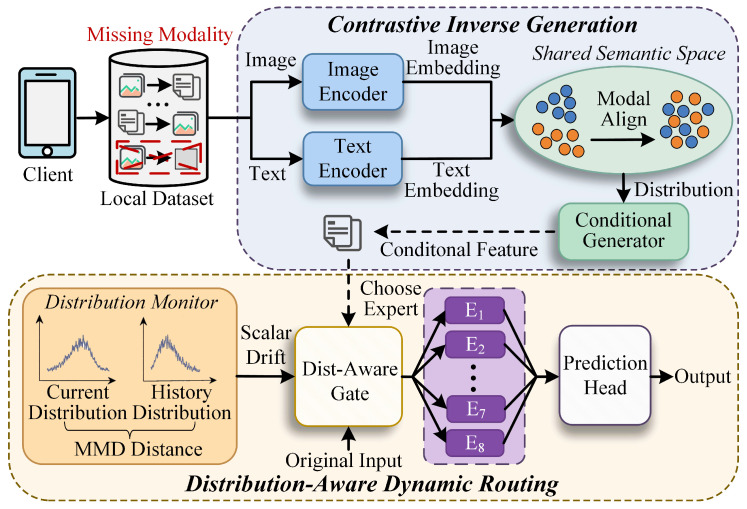
Overview of the FedMIR framework. Each client first passes its local multimodal data, which may have missing modalities, through modality-specific encoders to obtain image and text embeddings. In the CIG module, embeddings are aligned into a shared semantic space via a contrastive objective, and a conditional generator infers missing modality representations from the observed ones, producing a complete conditional feature. This representation is then forwarded to the DADR module, where a distribution monitor computes the MMD distance between current and historical feature distributions to generate a scalar drift signal. A distribution-aware gate combines this signal with the original input to dynamically select and weight the appropriate experts, and the aggregated expert output is sent to the retrieval head for the final output.

We propose a multimodal federated learning framework named FedMIRto address missing modalities and distribution shift in IoT deployments. Given local multimodal samples on each client, where some modalities may be unavailable, FedMIR first constructs a complete multimodal representation for each sample and then routes it to suitable experts according to the current distribution state to obtain the final retrieval output. Specifically, FedMIR consists of two consecutive steps, namely, contrastive inverse generation (CIG) and distribution-aware dynamic routing (DADR), as illustrated in Figure 2.

### 4.1. Design of Contrastive Inverse Generation

Since encoders trained in a shared semantic space are expected to produce similar representations for the same semantic entity, the core idea of FedMIR is to establish cross-modal relations by comparing the similarity between representations from different modalities. Although this idea is conceptually simple, directly comparing raw features across modalities is impractical because their feature dimensions and distributions differ and they cannot be aligned in a straightforward manner. Nevertheless, the semantic information conveyed by different modalities should be consistent. This implies that representations of the same entity from different modalities should be close in the shared semantic space.

Motivated by this observation, we employ contrastive learning to map heterogeneous modalities into a shared semantic space, where representations of paired samples are pulled together and representations of mismatched samples are pushed apart. Building on insights from contrastive representation learning, we find that cross-modal semantic associations can be established through this mechanism, and the representation of a missing modality can be naturally viewed as a conditional distribution given the observed modalities.

To obtain a shared semantic space aligned with the downstream retrieval objective, we design a multimodal contrastive alignment approach specialized for the image and text modalities considered in this paper. Their encoders are

$$E_{I}:X_{I}\to Z,\;E_{T}:X_{T}\to Z,$$

where Z is the shared semantic space. For a paired image–text sample $\left(x_{I},x_{T}\right)$, we obtain latent representations

$$z_{I}=E_{I}\left(x_{I}\right),\;z_{T}=E_{T}\left(x_{T}\right).$$

We adopt a bidirectional InfoNCE style contrastive objective to align the two modalities. Given a mini batch of size *B*, the alignment loss is

(8) $$L_{\mathrm{align}}=\frac{1}{2}\left(L_{I\to T}+L_{T\to I}\right),$$

where

(9) $$L_{I\to T}=-\frac{1}{B}\sum_{i=1}^{B}\log\frac{\exp\left(\mathrm{sim}(z_{i}^{I},z_{i}^{T})/\tau \right)}{\sum_{j=1}^{B}\exp\left(\mathrm{sim}(z_{i}^{I},z_{j}^{T})/\tau \right)},$$

and

(10) $$L_{T\to I}=-\frac{1}{B}\sum_{i=1}^{B}\log\frac{\exp\left(\mathrm{sim}(z_{i}^{T},z_{i}^{I})/\tau \right)}{\sum_{j=1}^{B}\exp\left(\mathrm{sim}(z_{i}^{T},z_{j}^{I})/\tau \right)},$$

with sim(·,·) denoting cosine similarity and τ>0 the temperature parameter. This bidirectional objective is consistent with the image–text retrieval formulation in Section 3; it pulls representations of the same semantic entity closer across modalities and pushes apart mismatched image–text pairs, thereby forming an aligned semantic structure in Z.

After alignment, different modalities share a similar distribution in Z but are not exactly identical. To infer the representation of a missing modality from an observed modality, CIG explicitly models their conditional dependence.

Let o∈{I,T} denote the observed modality and m∈{I,T}∖{o} the missing modality. For a complete pair, we can obtain $z_{m}=E_{m}\left(x_{m}\right)$ in the shared space. Given the representation $z_{o}$ of the observed modality, we model the latent representation $z_{m}$ of the missing modality with a conditional Gaussian

(11) $$p_{\theta }\left(z_{m}|z_{o}\right)=N\left(\mu_{\theta }\left(z_{o}\right),\sigma_{\theta }^{2}\left(z_{o}\right)I\right),$$

where $\mu_{\theta }:Z\to Z$ and $\sigma_{\theta }^{2}:Z\to R_{+}$ are neural networks with parameters θ. The mean $\mu_{\theta }$ captures the expected cross-modal mapping, while the variance $\sigma_{\theta }^{2}$ represents uncertainty. For modality pairs that are strongly correlated, the predicted variance is small and the generated representations are more stable.

We estimate the conditional distribution parameters by maximizing the log likelihood of complete pairs $\left(z_{o},z_{m}\right)$. The negative log-likelihood loss is

(12) $$L_{\mathrm{gen}}=-E_{\left(z_{o},z_{m}\right)}\left[\log p_{\theta }\left(z_{m}|z_{o}\right)\right],$$

which is equivalent to a variance-weighted mean squared error when (11) is Gaussian.

At inference time, for a sample with only modality *o*, we first compute

(13) $$z_{o}=E_{o}\left(x_{o}\right).$$

We then sample the missing modality representation in the semantic space

(14) $${\hat{z}}_{m}∼p_{\theta }\left(z_{m}|z_{o}\right)=N\left(\mu_{\theta }\left(z_{o}\right),\sigma_{\theta }^{2}\left(z_{o}\right)I\right),$$

We implement this sampling through the reparameterization trick

(15) $${\hat{z}}_{m}=\mu_{\theta }\left(z_{o}\right)+\sigma_{\theta }\left(z_{o}\right)⊙\varepsilon ,\;\varepsilon ∼N\left(0,I\right),$$

where ⊙ denotes element-wise multiplication. If needed, the latent representation can be decoded back to the data space through a decoder $D_{m}$ as

(16) $${\hat{x}}_{m}=D_{m}\left({\hat{z}}_{m}\right).$$

Through this procedure, clients with incomplete modalities can obtain approximately complete multimodal representations $\left(z_{o},{\hat{z}}_{m}\right)$ or reconstructed data $\left(x_{o},{\hat{x}}_{m}\right)$. The downstream model thus operates on a unified input format even when raw observations are missing.

Under the FL protocol, updates to the global conditional generator are restricted to clients with complete image–text pairs, while modality-incomplete clients are excluded from optimization. These clients synchronize the global generator from the server and use it only for inference; given an observed modality representation $z_{o}$, they sample the missing representation ${\hat{z}}_{m}$ via the reparameterization process before proceeding to the DADR module. The proportion of complete-pair clients participating in each round is 1−ρ, where ρ denotes the missing ratio. Under settings where ρ≤60%, at least 40% of sampled clients contribute to generator training per round. At the end of each communication round, the server aggregates generator parameters from complete-pair clients using sample size-weighted FedAvg and broadcasts the updated global model to all clients for the next iteration.

### 4.2. Design of Distribution-Aware Dynamic Routing

Mixture-of-experts architectures improve robustness under distribution shift by combining experts that specialize in different regions of the domain space. In IoT systems, however, the data distribution varies across clients and drifts over time as environments, devices, and user behaviors change. Routing networks that adapt only through gradient updates are often unable to respond to such changes with sufficient speed and stability. DADR mitigates this limitation by injecting an explicit distribution state into the gating mechanism and by adjusting the number of active experts according to the observed drift.

We design a lightweight local monitor to track distribution changes on each client. Let $D_{t}$ and $D_{t-\delta }$ denote feature distributions at time *t* and an earlier time t−δ. We define the drift strength as

(17) $$\Delta_{d}^{\left(t\right)}=\mathrm{MMD}\left(D_{t},D_{t-\delta }\right)+\lambda |\sigma_{t}^{2}-\sigma_{t-\delta }^{2}|,$$

where $\sigma_{t}^{2}$ is the within-class variance at time *t* and λ is a non-negative weight. The first term captures global distribution changes and the second term captures changes in local variance.

To compute the maximum mean discrepancy between two feature distributions, we use the standard unbiased empirical estimator. Given mini batches $X_{t}={\left\{h_{i}^{t}\right\}}_{i=1}^{n_{t}}$ sampled from $D_{t}$ and $X_{t-\delta }={\left\{h_{j}^{t-\delta }\right\}}_{j=1}^{n_{t-\delta }}$ sampled from $D_{t-\delta }$, the MMD is

(18) $$\begin{array}{cc} \mathrm{MMD}\left(D_{t},D_{t-\delta }\right)\; & =\frac{1}{n_{t}\left(n_{t}-1\right)}\sum_{i\neq j}k\left(h_{i}^{t},h_{j}^{t}\right)+\frac{1}{n_{t-\delta }\left(n_{t-\delta }-1\right)}\sum_{i\neq j}k\left(h_{i}^{t-\delta },h_{j}^{t-\delta }\right)\\ \; & \;-\frac{2}{n_{t}n_{t-\delta }}\sum_{i=1}^{n_{t}}\sum_{j=1}^{n_{t-\delta }}k\left(h_{i}^{t},h_{j}^{t-\delta }\right), \end{array}$$

where k(·,·) is a positive definite kernel such as the Gaussian radial basis function.

To reduce noise and capture long-term trends, we smooth the drift strength by exponential moving average

(19) $$s_{d}^{\left(t\right)}=\alpha \Delta_{d}^{\left(t\right)}+\left(1-\alpha \right)s_{d}^{\left(t-1\right)},$$

where $s_{d}^{\left(t\right)}$ is the scalar distribution state at time *t* and α∈(0,1) controls the trade-off between current and historical observations.

A standard gating network in MoE uses only the current input feature *x* to compute expert selection probabilities

(20) $$g\left(x\right)=\mathrm{Softmax}\left(W_{g}x\right),$$

where $W_{g}$ is a learnable matrix. This design ignores distribution-level information. DADR extends the gate so that it takes both the input and the distribution state as evidence:

(21) $$g\left(x,s_{d}\right)=\mathrm{Softmax}\left(W_{g}\left[x;\phi \left(s_{d}\right)\right]\right),$$

where $\left[x;\phi \left(s_{d}\right)\right]$ denotes concatenation and $\phi :R\to R^{d_{s}}$ encodes the scalar state into a low-dimensional vector. We use a simple linear projection followed by a ReLU activation

(22) $$\phi \left(s_{d}\right)=\mathrm{ReLU}\left(W_{s}s_{d}+b_{s}\right),$$

which introduces only a small number of additional parameters while allowing the gate to adapt its decisions according to distribution dynamics.

In addition to modifying the probability of each expert, DADR also adjusts the number of activated experts. Intuitively, a strong distribution shift requires more experts to cover new patterns, while a stable distribution can be handled with a small set of experts to save computation. Let *K* be the base number of activated experts, $\tau_{\mathrm{drift}}$ be a drift threshold, and *N* be the total number of experts. We define the dynamic number of active experts as

(23) $$K^{\prime }=\left\{\begin{array}{cc} K+\left\lfloor \gamma |s_{d}|\right\rfloor , & |s_{d}|>\tau_{\mathrm{drift}},\\ K, & \mathrm{otherwise}, \end{array}\right.$$

where γ>0 controls the growth rate. When the absolute distribution state $|s_{d}|$ increases, the system gradually activates more experts in proportion to the drift level, which allocates computation resources according to need.

Given an input feature *x*, state $s_{d}$, and expert set $\left\{E_{1},\ldots ,E_{N}\right\}$, the routing procedure is as follows: We first compute distribution-aware scores by (21). We then select the top $K^{\prime }$ experts according to these scores, renormalize the selected scores by a Softmax operation, and take a weighted sum of the corresponding expert outputs. This yields the final routed representation used by the downstream task head.

In federated settings, persistent distribution shift can cause systematic bias in expert usage across clients. Some experts may be heavily loaded while others are rarely used, which harms the robustness of the MoE architecture. To avoid this imbalance, we add a load-balancing term to the global objective

(24) $$L_{\mathrm{balance}}=\sum_{j=1}^{N}{\left(\frac{1}{N}-{\bar{f}}_{j}\right)}^{2},$$

where ${\bar{f}}_{j}$ is the average activation frequency of expert *j* over all clients. This term encourages a more uniform utilization of experts and prevents structural degeneration of the MoE.

More concretely, at each communication round *t*, every participating client $c\in S_{t}$ computes a local expert activation frequency vector $f^{\left(c\right)}$, whose *j*-th element is $f_{j}^{\left(c\right)}$, and uploads it together with the model parameter update. The server then aggregates these statistics using sample size weights,

$${\bar{f}}_{j}=\sum_{c\in S_{t}}\frac{n_{c}}{N_{t}}f_{j}^{\left(c\right)},\;blueN_{t}=\sum_{c\in S_{t}}n_{c},$$

and broadcasts the aggregated vector $\bar{f}$ to all clients for computing the load-balancing loss in the next round. Under the federated learning framework, each client transmits only its local model parameter updates $\Delta \Theta^{\left(c\right)}$ and an *N*-dimensional activation frequency vector $f^{\left(c\right)}$, ensuring that sensitive local data remain on-device. Accordingly, the privacy leakage risk remains low because FedMIR uploads only model parameter updates together with aggregated routing statistics, rather than raw features or original multimodal samples.

### 4.3. Optimizing Objects

The overall training objective is a weighted sum of task and regularization losses

(25) $$L_{\mathrm{total}}=L_{\mathrm{task}}+\alpha L_{\mathrm{align}}+\beta L_{\mathrm{balance}}+\gamma L_{\mathrm{gen}},$$

where $L_{\mathrm{task}}$ is the downstream bidirectional image–text retrieval loss defined in (5). The terms $L_{\mathrm{align}}$, $L_{\mathrm{gen}}$, and $L_{\mathrm{balance}}$ are the contrastive alignment loss in (8), the generation loss in (12), and the load-balancing loss in (24). Scalars α, β, and γ control their relative strengths.

Training follows the standard federated protocol with alternating local updates and global aggregation. During local updates, each client *c* performs several gradient steps on its dataset $D_{c}$. Clients with incomplete modalities first apply CIG to recover semantic representations of missing modalities, then apply DADR for distribution-aware expert routing, and finally compute the local loss $L_{\mathrm{total}}$ to update the local parameters. All CIG reconstructions and DADR decisions are performed strictly on the client side; the recovered representations ${\hat{z}}_{m}$ never leave the device. During global aggregation, the server collects only model parameter updates from selected clients, computes a weighted average such as FedAvg, and updates the global model. The server also gathers aggregated, scalar statistics of expert activations to estimate ${\bar{f}}_{j}$ in (24), which is used to refine the load-balancing term in subsequent rounds. Expert activation counts are accumulated over many local samples before being uploaded, so no sample-level representation or label information is revealed.

Algorithm 1 summarizes the training and inference procedures of FedMIR in a more compact and formal manner, emphasizing the interaction between CIG, DADR, and federated aggregation.
**Algorithm 1** FedMIR: Federated Training and Inference     **Input:** data partitions ${\left\{D_{c}\right\}}_{c=1}^{C}$ and initial global parameters $\Theta^{0}$, communication rounds *R*, local epochs *E*, and base expert number *K*     **Output:** trained parameters $\Theta^{R}$  1:**for** t=0 to R−1 **do**  2:      Sample participating clients $S_{t}\subseteq C$ and broadcast $\Theta^{t}$  3:      **for all** $c\in S_{t}$ **in parallel do**  4:         Set $\Theta_{c}^{t}\leftarrow \Theta^{t}$  5:         **for** e=1 to *E* **do**  6:               **for all** $B_{c}∼D_{c}$ **do**  7:                   Encode available modalities into the shared space: $\left(z^{I},z^{T}\right)$  8:                   Compute $L_{\mathrm{align}}$ by Equation (8)  9:                   **if** a modality is missing **then**10:                      Infer ${\hat{z}}_{m}∼p_{\theta }\left(z_{m}|z_{o}\right)$ and set $\tilde{z}\leftarrow \left[z_{o};{\hat{z}}_{m}\right]$11:                   **else**12:                      Set $\tilde{z}\leftarrow \left[z^{I};z^{T}\right]$13:                   **end if**14:                   Update $s_{d}^{\left(t\right)}$ using Equations (17)–(19)15:                   Compute $K^{\prime }$ by Equation (23) and route $\tilde{z}$ with $g(\tilde{z},s_{d}^{\left(t\right)})$16:                   Evaluate $L_{\mathrm{task}}$ by Equation (5) and $L_{\mathrm{total}}$ by Equation (25)17:                   Update $\Theta_{c}^{t}\leftarrow \Theta_{c}^{t}-\eta \;\nabla L_{\mathrm{total}}$18:              **end for**19:         **end for**20:         Upload local parameters and auxiliary statistics to the server21:      **end for**22:      Aggregate client updates with FedAvg to obtain $\Theta^{t+1}$23:**end for**

## 5. Experiment and Result

### 5.1. Experiment Settings

#### 5.1.1. Dataset

We evaluate FedMIR on both Flickr30k and MS-COCO. **Flickr30k** [35] contains 31,783 images collected from Flickr, each annotated with five human-written English captions, with 29,783 images for training, 1000 for validation, and 1000 for testing. **MS-COCO** [36] comprises 123,287 images from the Microsoft Common Objects in Context dataset, each paired with five reference captions. We adopt the Karpathy split [37], using 113,287 images for training, 5000 for validation, and 5000 for testing. We report results on the 5-fold 1K setting, following [38]. Because no publicly available multimodal IoT dataset currently supports large-scale federated evaluation, we use image–text benchmarks as a practical proxy for two of the most common information sources in IoT deployments, namely, visual observations and textual metadata. To better reflect heterogeneous IoT sensing conditions, we further simulate diverse modality configurations and missing data patterns following the evaluation protocol of DreamLIP [39], as detailed in Section 5.1.4.

#### 5.1.2. Metrics

Our primary evaluation metric is Recall at K (R@K). We report R@1 and R@5 for both image-to-text (i2t) and text-to-image (t2i) retrieval directions. R@1 reflects the model’s ability to rank the correct match first, while R@5 measures whether at least one correct match appears in the top five results, indicating the model’s ability to provide a useful candidate set in practical retrieval. Additionally, we report the mean of all four metrics as the Rmean score, summarizing cross-modal retrieval performance.

#### 5.1.3. Baselines

We compare FedMIR against eleven representative federated learning methods. To ensure a fair comparison, all baselines employ the same ViT-Small image encoder [40] and BERT-base text encoder [41] as FedMIR, thereby maintaining identical parameter budgets across methods. Baselines originally designed for classification tasks are adapted to the image–text retrieval task by replacing their classification head with the same bidirectional contrastive retrieval objective defined in Equation (5), and by applying the same data partitioning and missing modality simulation protocol described in Section 5.1.4. For all baselines, hyperparameters are tuned on the validation set using the same search budget as FedMIR.

- FedAvg [42]: Server aggregates client parameters by weighted averaging.
- FedProx [43]: Adds a proximal term to the local objective to limit client drift.
- FedMD [15]: Distills predictions on a public dataset.
- FedGEMS [17]: Fuses client knowledge to train a global model and distills it back.
- FedIoT [44]: Trains modality-specific autoencoders and aggregates representations.
- FedCG [45]: Replaces raw data exchange with synthetic features.
- FedCLIP [39]: Introduces an attention-based adapter on a pretrained CLIP model.
- CreamFL [38]: Ensembles client representations using a contrastive objective.
- FedCola [20]: FL via parameter sharing and knowledge exchange.
- MFL-AKD [46]: Speeds up stragglers through knowledge transfer by distillation.
- FedVMR [19]: Enables federated training for video–text moment retrieval.

#### 5.1.4. Implementations

Following the implementation settings of FedCola [20], we adopt ImageNet-pretrained ViT-Small [40] as the image encoder and BERT-base [41] as the text encoder, mapping both to a shared semantic space of dimension d=512. Images are resized to 224×224 pixels and texts are tokenized with a maximum length of 40. The conditional generator is a three-layer MLP with a hidden dimension of 1024 and ReLU activations, using a contrastive temperature τ=0.07. For the DADR module, we employ N=8 experts with K=2 initially activated, setting the drift threshold as $\tau_{\mathrm{drift}}=0.05$, the growth rate as γ=2.0, and the EMA momentum as α=0.9. Loss weights are configured as α=1.0 for alignment, β=0.01 for load balancing, and λ=0.5 for generation.

To simulate real-world conditions, data are partitioned across 20 clients using a Dirichlet distribution (α=0.5), with 50% of clients sampled per round. Missing modality ratios ρ∈{0%,20%,40%,60%} are introduced by randomly assigning a fixed fraction of clients to lack one modality. Temporal distribution shift is further emulated by dividing local data into T=5 consecutive time windows with shift intensity δ∈{0.0,0.1,0.2,0.3}. The corresponding distribution skew is controlled via $\delta_{\mathrm{dir}}=1/\left(1+10\delta \right)$, where the reference for drift detection in Equation (17) is the immediately preceding window. Federated training is conducted for 100 rounds with E=5 local epochs, a learning rate of $1\times 10^{-4}$, and the AdamW optimizer. Global aggregation is performed via FedAvg on a workstation equipped with an Intel Xeon Gold 6430 CPU and an NVIDIA RTX 4090 GPU.

### 5.2. Main Results

Table 2 presents the cross-modal retrieval results obtained when all clients have access to both image and text modalities, averaged over five independent runs with standard deviation reported. FedMIR achieves the best performance across all metrics on both datasets, with an Rmean of 76.1, outperforming MFL-AKD by 2.9%. On Flickr30k, FedMIR improves i2t R@1 by 2.3% and t2i R@1 by 2.5% compared to FedCola. On the more challenging MS-COCO dataset, it achieves a 1.6% improvement in t2i R@1 over MFL-AKD. FedMIR’s CIG module establishes stronger cross-modal correspondences than representation averaging or simple distillation. Compared with FedCola, which lacks distribution-aware mechanisms, the consistent performance gap of approximately 7.4% in Rmean demonstrates that explicitly modeling distribution states through DADR enhances robustness under heterogeneous non-IID data. The mixture-of-experts backbone with dynamic routing further enables FedMIR to adaptively handle varying data characteristics across clients. The statistical significance of the performance differences between FedMIR and each baseline is evaluated using a two-sided paired *t*-test on Rmean with Bonferroni correction for 11 comparisons, resulting in a threshold of $\alpha^{\prime }=0.01/11\approx 9\times 10^{-4}$. Even the two strongest baselines, MFL-AKD and FedVMR, exhibit noticeable performance gaps relative to FedMIR, along with higher run-to-run variance, whereas FedMIR remains consistently stable across runs. Consequently, the superiority of FedMIR over all evaluated baselines is statistically significant at $p<9\times 10^{-4}$.

**Figure 3:**
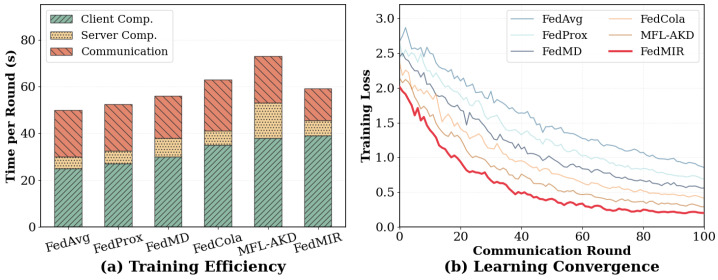
Per-round time cost and convergence speed.

Figure 3 breaks down the computational cost per communication round for different methods, averaged per round on MS-COCO with 20 clients. FedMIR’s client computation time is 56% higher than that of FedAvg. This overhead mainly comes from contrastive alignment and conditional generation for missing modalities, as well as distribution state estimation and dynamic routing. In terms of communication, FedMIR follows the same parameter-sharing protocol as FedAvg and does not transmit any features, embeddings, or raw samples; both methods exchange only model parameter updates between clients and the server. FedMIR nevertheless reduces the per-round communication payload by about 32% relative to FedAvg because the mixture-of-experts backbone is sparsely activated and each client only needs to upload updates for the experts and gating components touched during its local training, together with the shared encoders and task head, rather than a full, dense network of comparable capacity. FedMIR’s server computation is modest compared to that of MFL-AKD, which performs centralized knowledge distillation. FedMIR’s server mainly performs aggregation and global model updates, with minimal additional overhead from collecting expert activation statistics for load balancing. Although FedMIR has a higher per-round cost, its faster convergence reduces the total number of rounds needed.

### 5.3. Ablation Study

#### 5.3.1. Impact of Different Module

Table 3 presents results when removing major components of FedMIR. Removing the entire CIG module causes a 14.5% degradation. Without conditional generation, the model resorts to simple mean imputation for missing modalities, failing to capture the semantic content of the absent data. Removing contrastive alignment while keeping the conditional generator results in 58.2% performance. This 3.4% gap suggests that the generator alone provides some benefit, but its effectiveness depends on well-aligned modalities in the shared semantic space. Removing the entire DADR module reduces performance by 5.6%. The standard MoE baseline can specialize experts but cannot adapt to distribution drift, because its gating decisions rely solely on input features, not on distributional context. Fixing the number of activated experts leads to a 3.4% degradation, revealing that optimal expert utilization varies with distribution stability. FedMIR’s dynamic K adaptation automatically adjusts this trade-off. Without load balancing, over extended training, expert usage becomes skewed, with 2–3 experts handling >70% of samples while others remain underutilized. The load-balancing term maintains expert diversity.

#### 5.3.2. Impact of Missing Modality Ratios ρ

Table 4 presents retrieval performance under varying missing modality ratios ρ∈{20%,40%,60%}. Performance degrades sharply as missing ratios increase. At ρ=60%, traditional FL methods lose nearly 40% of their original performance, while knowledge distillation methods suffer a drop beyond 25%. These methods lack mechanisms to handle incomplete inputs, with clients missing modalities either failing to produce meaningful representations or generating erroneous predictions that contaminate global aggregation. FedMIR retains 83.7% of its original performance even at ρ=60%, with only a 7.5% average performance drop across all missing ratios. This robustness stems directly from CIG’s conditional generation mechanism: by learning the conditional distribution in the aligned semantic space, FedMIR reconstructs plausible representations for missing modalities, preserving semantic consistency for effective retrieval. Surprisingly, CreamFL experiences a 20.4% average drop under missing modalities, worse than even simple FedAvg. CreamFL’s contrastive aggregation requires comparing representations from both modalities to compute alignment scores, but this mechanism breaks down when clients have only one modality. Without paired representations, contrastive weighting produces unreliable aggregation weights, leading to poor global model quality.

**Figure 4:**
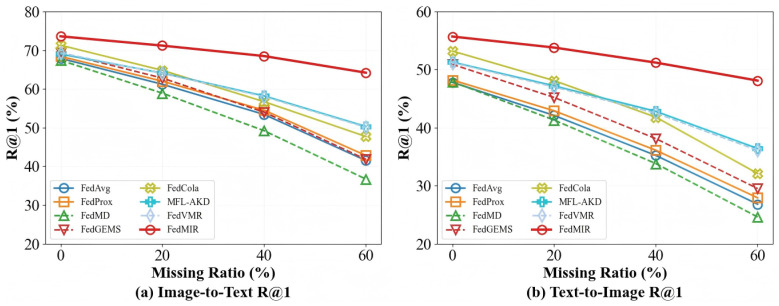
R@1 performance by retrieval direction under varying missing ratios on MS-COCO.

Figure 4 shows the performance across retrieval directions under different missing ratios on MS-COCO. t2i experiences more severe degradation than i2t across all methods. For example, FedCola’s t2i R@1 drops 39.6% relative at ρ=60%, while i2t R@1 drops 33.0% relative. This asymmetry arises because ViTs have larger parameter capacity and learn richer representations than text encoders, making the absence of visual information more detrimental to retrieval quality. FedMIR maintains more symmetric performance across directions, indicating that CIG’s conditional generation is equally effective at inferring missing text from images and vice versa, demonstrating the generality of the learned cross-modal conditional distributions.

#### 5.3.3. Impact of Different Heterogeneity Levels

**Figure 5:**
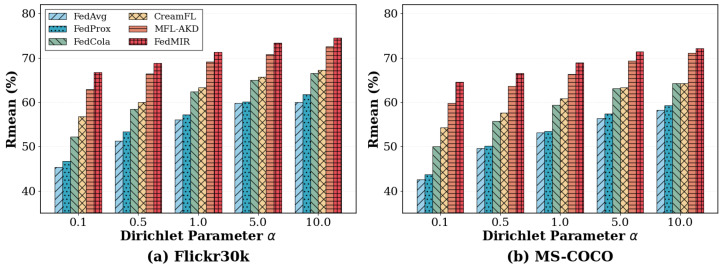
Performance under different heterogeneity levels on MS-COCO with ρ=30%.

Figure 5 examines performance under varying degrees of data heterogeneity controlled by the Dirichlet parameter α. FedMIR consistently maintains superior performance across all heterogeneity levels. As non-IID severity increases, the performance gap between methods becomes more pronounced: under high heterogeneity, the performance variance across methods reaches 19.7%, while, under near-IID settings, it reduces to 11.9%. This indicates that poorly designed aggregation and alignment mechanisms are exposed under severe non-IID conditions. CreamFL is particularly sensitive to α: its performance increases by 19.0% when moving from α=0.1 to α=10.0, compared to FedMIR’s 11.9% increase. This suggests that contrastive representation aggregation alone, without proper missing modality handling and distribution awareness, struggles under severe client heterogeneity due to the difficulty of establishing meaningful similarity comparisons when client representations are drawn from highly divergent distributions.

#### 5.3.4. Impact of the Number of Clients

**Figure 6:**
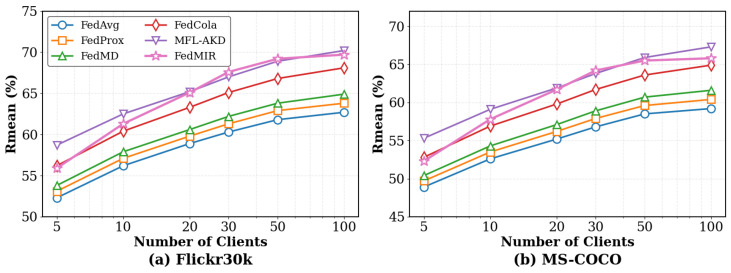
Performance vs. number of clients on MS-COCO (ρ=30%, α=0.5).

Figure 6 shows how performance scales with the number of participating clients. As the number of clients increases from 5 to 100, all methods improve, confirming that federated learning can aggregate knowledge from more data sources. FedMIR exhibits steeper scaling than FedAvg, indicating more efficient knowledge aggregation. Specifically, at 5 clients, FedMIR outperforms FedAvg by 6.9%, and, at 100 clients, this gap widens to 11.1%. This suggests that FedMIR’s cross-modal alignment and distribution-aware routing become increasingly effective as more diverse client knowledge becomes available. For all methods, the marginal benefit of adding clients decreases beyond 50 clients, as the global data distribution is already well covered and communication/coordination overhead grows with more participants.

#### 5.3.5. Impact of Different Distribution Shift Intensities δ

**Figure 7:**
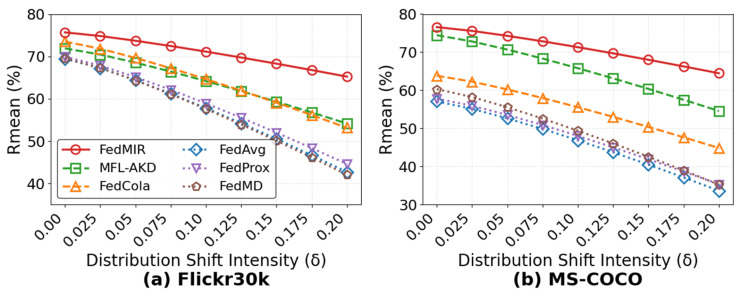
Robustness to distribution shift across methods.

Figure 7 reports performance under different distribution shift intensities. Under the strongest shift, FedMIR retains 84.2% of its no-shift performance, while even advanced baselines such as FedCola and MFL-AKD retain 70.3% and 73.3%, respectively. This robustness stems from FedMIR’s distribution state estimation via MMD and variance tracking. By continuously monitoring the distribution state $s_{d}$ with temporal smoothing, FedMIR detects drift in real time and triggers the dynamic *K* mechanism to activate additional experts proactively. For instance, when δ increases from 0.0 to 0.2, the average $s_{d}$ across clients rises from 0.02 to 0.18, which increases the number of activated experts and prevents performance collapse.

## 6. Discussion

Table 5 provides empirical support for the methodological analysis developed in Section 2. Specifically, the consistently weak performance of discard, zero-fill, and mean-fill is consistent with the limitations of late fusion paradigms under incomplete local observations, where degraded supervision and non-informative inputs can exacerbate prediction inconsistency across clients. Likewise, the limited gains of nearest-neighbor and other simple recovery strategies reflect a key limitation of intermediate fusion methods: directly combining heterogeneous or crudely completed representations is insufficient to resolve cross-modal distribution mismatch and may instead intensify knowledge conflicts. The persistent gap between CIG and raw-space generative baselines such as VAE and GAN further suggests that hierarchical fusion or expert-weighting mechanisms alone remain inadequate when the missing modality is not first reconstructed in a semantically aligned space. These findings provide a clearer empirical basis for the comparisons that follow. The consistent benefit of DADR over standard MoE baselines corroborates findings from FedDG [34] and DFLMoE [33], showing that domain-aware gating improves generalization under distribution shifts while extending these approaches to the federated setting, where shift signals must be estimated locally without access to global data.

**Figure 8:**
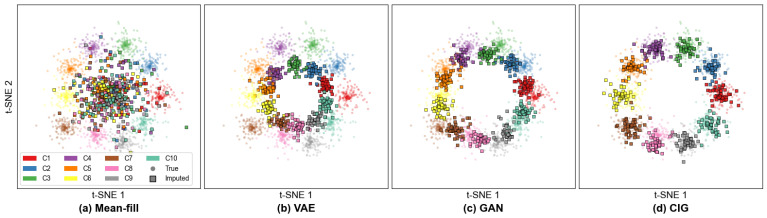
t-SNE visualization comparing recovered text representations.

The t-SNE visualization in Figure 8 shows that mean-fill produces a single dense cluster regardless of class labels, indicating a collapse of semantic structure. Nearest-neighbor imputation requires storing and searching over the full training set, introducing privacy risks and computational overhead. VAE- and GAN-based methods improve over simple imputation by generating plausible modality features. The VAE achieves 61.4%, while the GAN reaches 62.7% through adversarial training; however, both methods generate modalities independently without explicit cross-modal conditioning and operate in the original high-dimensional data space. In contrast, FedMIR’s CIG outperforms all alternatives by 6.6%. The key insight is operating in an aligned semantic space rather than the raw data space. After contrastive alignment, the conditional distribution becomes significantly simpler because semantically similar images and texts are mapped to nearby regions. As a result, a lightweight conditional Gaussian model is sufficient to capture the structure of the missing modality.

## 7. Conclusions

In this paper, we presented FedMIR, a novel framework for multimodal federated learning under missing modalities and distribution shift. FedMIR leverages cross-modal semantic consistency to recover missing modality representations through CIG and employs distribution state estimation to enable adaptive expert routing via DADR. Throughout training and inference, FedMIR strictly follows the parameter-sharing federated protocol: missing modality reconstructions and routing decisions are performed locally, and only model parameter updates, together with aggregated expert-usage statistics, are exchanged with the server. Extensive experiments on the Flickr30k and MS-COCO benchmarks demonstrate that the cross-modal alignment learned by CIG effectively reconstructs missing modality representations while preserving semantic relationships required for downstream tasks. The current framework is instantiated and evaluated in the bimodal image–text setting, which represents the most widely studied configuration in multimodal learning. Taken together, these findings suggest that FedMIR provides a useful foundation for exploring the interplay between cross-modal semantic consistency and distribution adaptation in federated learning. In future work, we will evaluate FedMIR on real-world IoT datasets with native sensor heterogeneity, missing modalities, and evolving data distributions to assess its practical effectiveness beyond benchmark image–text corpora. We will also extend the framework to richer multimodal settings, such as IoT systems integrating visual, audio, and other sensor streams, and incorporate continual learning mechanisms that incrementally update the conditional generator and routing strategy without causing catastrophic forgetting.

## Figures and Tables

**Table 1:** Comparison of different MFL method categories. ✓ indicates the property is fully supported, ✗ indicates not supported, and ∘ indicates partially supported.

Property	Late	Intermediate	Hierarchical	MoE	FedMIR
Missing modality recovery	✗	✗	∘	✗	✓
Distribution shift adaptation	✗	✗	✗	∘	✓
Cross-modal semantic alignment	✗	✓	✓	✗	✓
Privacy preservation	✓	∘	✓	✓	✓
Dynamic expert routing	✗	✗	✗	✓	✓
Distribution-aware gating	✗	✗	✗	✗	✓

**Table 2:** Cross-modal retrieval performance on Flickr30k and MS-COCO with complete modalities. Best results in **bold**, second-best per column underlined.

Method	Flickr30k				MS-COCO				Rmean	Sig.
	i2t		t2i		i2t		t2i			
	R@1	R@5	R@1	R@5	R@1	R@5	R@1	R@5		
ine FedAvg	67.9±0.6	89.0±0.4	47.8±0.7	73.1±0.5	45.2±0.6	76.7±0.5	34.7±0.7	71.7±0.5	63.3±0.6	p<0.001
FedProx	68.4±0.5	89.3±0.4	48.2±0.6	73.8±0.5	46.1±0.5	77.4±0.4	35.3±0.6	72.4±0.5	63.9±0.5	p<0.001
FedMD	67.4±0.7	89.1±0.5	47.9±0.8	73.8±0.6	48.4±0.7	80.2±0.5	38.2±0.8	74.4±0.6	64.9±0.7	p<0.001
FedGEMS	69.4±0.6	90.7±0.4	50.9±0.5	74.5±0.5	48.7±0.6	80.5±0.4	38.7±0.6	74.8±0.5	66.0±0.5	p<0.001
FedIoT	67.2±0.8	88.7±0.6	45.4±0.9	71.9±0.7	43.3±0.8	75.6±0.6	33.9±0.9	70.1±0.7	62.0±0.8	p<0.001
FedCG	66.5±0.9	87.8±0.7	44.9±1.0	70.4±0.8	63.8±0.7	88.5±0.5	45.8±0.8	78.7±0.6	68.3±0.7	p<0.001
FedCLIP	70.2±0.5	91.4±0.4	52.1±0.5	76.3±0.4	50.3±0.5	82.1±0.4	40.6±0.5	76.9±0.4	67.5±0.5	p<0.001
CreamFL	69.7±0.6	90.9±0.5	51.4±0.6	75.6±0.5	49.7±0.6	80.7±0.5	38.9±0.7	75.0±0.5	66.5±0.6	p<0.001
FedCola	$\underline{71.3\pm 0.4}$	$\underline{92.1\pm 0.3}$	$\underline{53.2\pm 0.5}$	$\underline{77.4\pm 0.4}$	51.8±0.5	83.4±0.4	41.9±0.5	78.2±0.4	68.7±0.4	p<0.001
MFL-AKD	69.0±0.8	91.5±0.6	51.3±0.9	75.8±0.7	66.1±0.7	$\underline{94.2\pm 0.5}$	$\underline{54.4\pm 0.8}$	$\underline{83.1\pm 0.6}$	$\underline{73.2\pm 1.0}$	p<0.01
FedVMR	69.3±0.8	91.3±0.6	51.2±0.8	75.2±0.7	$\underline{66.3\pm 0.7}$	94.1±0.5	54.2±0.8	82.8±0.6	73.1±0.9	p<0.01
ine **FedMIR**	**73.6** ± **0.3**	**93.8** ± **0.2**	**55.7** ± **0.3**	**79.6** ± **0.3**	**68.4** ± **0.3**	****95.7** ± **0.2****	**56.8** ± **0.3**	**85.4** ± **0.3**	**76.1** ± **0.3**	-

**Table 3:** Ablation study on Flickr30k and MS-COCO with ρ=40% missing ratio. Best results in **bold**. Δ indicates a performance drop compared to the full model.

Method	Flickr30k				MS-COCO				Rmean	ΔRmean
	i2t		t2i		i2t		t2i			
	R@1	R@5	R@1	R@5	R@1	R@5	R@1	R@5		
ine **FedMIR** (full)	**70.8** ± **0.4**	**91.2** ± **0.3**	**53.4** ± **0.4**	**77.1** ± **0.3**	**65.9** ± **0.4**	**93.4** ± **0.3**	**54.2** ± **0.4**	**82.7** ± **0.3**	**69.3** ± **0.4**	−−−
*w*/*o* CIG	52.6±1.2	76.8±0.9	38.9±1.3	63.2±1.0	50.3±1.1	78.5±0.8	39.8±1.2	70.4±0.9	54.8±1.1	−14.5±1.2
*w*/*o* Alignment	56.2±1.0	80.4±0.8	42.3±1.1	66.8±0.9	53.7±0.9	82.1±0.7	43.2±1.0	73.9±0.8	58.2±0.9	−11.1±1.0
*w*/*o* Generator	59.4±0.8	83.6±0.6	45.8±0.9	69.7±0.7	57.1±0.8	85.8±0.6	46.5±0.8	76.2±0.7	60.5±0.8	−8.8±0.9
*w*/*o* DADR	61.3±0.7	85.7±0.5	47.9±0.7	71.4±0.6	59.8±0.7	88.2±0.5	48.9±0.7	78.6±0.6	63.7±0.7	−5.6±0.8
*w*/*o* Dynamic *K*	63.5±0.6	87.4±0.4	49.6±0.6	73.2±0.5	62.1±0.5	90.3±0.4	51.2±0.6	80.1±0.5	65.9±0.5	−3.4±0.6
*w*/*o* Load Balance	64.8±0.5	88.9±0.4	51.1±0.6	74.8±0.5	63.7±0.5	91.6±0.4	52.6±0.5	81.3±0.4	67.1±0.5	−2.2±0.6
Static Gating	62.1±0.7	86.5±0.5	48.7±0.7	72.3±0.6	61.5±0.6	89.4±0.5	49.8±0.7	79.4±0.5	64.5±0.6	−4.8±0.7
*w*/*o* Dist. State	60.8±0.8	85.1±0.6	47.2±0.8	70.8±0.7	60.2±0.7	87.9±0.5	48.3±0.8	77.8±0.6	63.0±0.7	−6.3±0.8
FedMIR-Lite	60.1±0.9	84.3±0.7	46.5±1.0	69.5±0.8	58.9±0.8	86.7±0.6	47.2±0.9	76.8±0.7	62.4±0.8	−6.9±0.9
Fixed MoE (K=2)	63.8±0.6	87.6±0.4	49.9±0.6	73.5±0.5	62.4±0.5	90.5±0.4	51.4±0.6	80.3±0.5	66.1±0.5	−3.2±0.6
Fixed MoE (K=4)	62.4±0.7	86.8±0.5	48.5±0.7	72.1±0.6	61.2±0.6	89.3±0.5	50.1±0.6	79.2±0.5	64.7±0.6	−4.6±0.7

**Table 4:** Impact of missing modality ratios on MS-COCO. Performance reported as Rmean (%).

Method	ρ=0%	ρ=20%	ρ=40%	ρ=60%	Avg. Drop
FedAvg	63.3±0.6	57.2±0.8	49.8±1.0	38.6±1.3	14.8±0.9
FedProx	63.9±0.5	58.1±0.7	50.9±0.9	39.7±1.2	14.3±0.8
FedMD	64.9±0.7	56.4±1.0	47.2±1.2	35.1±1.5	18.7±1.1
FedGEMS	66.0±0.5	58.9±0.8	50.4±1.0	38.9±1.3	16.6±0.9
FedIoT	62.0±0.8	54.3±1.0	45.6±1.2	33.8±1.5	17.4±1.1
FedCG	68.3±0.7	60.2±0.9	51.7±1.1	40.3±1.3	17.6±1.0
FedCLIP	67.5±0.5	59.8±0.8	51.2±1.0	39.8±1.2	17.2±0.9
CreamFL	66.5±0.6	57.1±1.0	46.9±1.3	34.2±1.6	20.4±1.2
FedCola	68.7±0.4	61.4±0.7	53.1±0.9	42.6±1.1	16.3±0.8
MFL-AKD	73.2±1.0	67.8±1.1	61.4±1.2	52.9±1.3	12.5±1.0
FedVMR	73.1±0.9	67.5±1.0	61.1±1.1	52.4±1.2	12.8±1.0
**FedMIR**	**76.1** ± **0.3**	**72.9** ± **0.4**	**69.3** ± **0.4**	**63.7** ± **0.5**	**7.5** ± **0.4**

**Table 5:** Comparison of missing modality recovery strategies on MS-COCO with ρ=40%.

Strategy	Rmean	i2t R@1	t2i R@1
Discard	47.3±1.4	38.6±1.6	29.4±1.5
Zero-fill	51.2±1.2	41.8±1.4	32.7±1.3
Mean-fill	54.8±1.0	44.5±1.2	35.1±1.1
Nearest-neighbor	58.6±0.8	47.9±1.0	38.2±0.9
VAE	61.4±0.7	50.3±0.9	40.6±0.8
GAN	62.7±0.7	51.6±0.8	41.9±0.8
CIG	69.3±0.4	63.2±0.5	54.7±0.5

## Data Availability

The source code used to generate the results presented in this paper is openly available for collaboration and development at the following GitHub repository: https://github.com/Leo1224-xhy/FedMIR/tree/master (accessed on 6 March 2026).
